# The morbidity and mortality following a diagnosis of peripheral arterial disease: Long-term follow-up of a large database

**DOI:** 10.1186/1471-2261-5-14

**Published:** 2005-06-22

**Authors:** Jaime Caro, Kristen Migliaccio-Walle, Khajak J Ishak, Irina Proskorovsky

**Affiliations:** 1 Caro Research Institute, Concord, MA, USA; 2 Division of General Internal Medicine, McGill University, Montreal, Quebec, Canada; 3 Caro Research, Montreal, Quebec, Canada

## Abstract

**Background:**

Awareness of the significance of peripheral arterial disease is increasing, but quantitative estimates of the ensuing burden and the impact of other risk factors remains limited. The objective of this study was to fill this need.

**Methods:**

Morbidity and mortality were examined in 16,440 index patients diagnosed with peripheral arterial disease in Saskatchewan, Canada between 1985 and 1995. Medical history and patient characteristics were available retrospectively to January 1980 and follow-up was complete to March 1998. Crude and adjusted event rates were calculated and Kaplan-Meier survival curves estimated. Cox proportional hazards analyses were conducted to examine the effect of risk factors on these rates. Patients suffering a myocardial infarction or ischemic stroke in Saskatchewan provided two reference populations.

**Results:**

Half of the index patients were male; the majority was over age 65; 73% had at least one additional risk factor at index diagnosis; 10% suffered a subsequent stroke, another 10% a myocardial infarction, and 49% died within the mean follow-up of 5.9 years. Annual mortality (8.2%) was higher among patients with PAD than after a myocardial infarction (6.3%) but slightly lower than that in patients suffering a stroke (11.3%). Index patients with comorbid disease (e.g., diabetes) were at highest risk of death and other events.

**Conclusion:**

A diagnosis of peripheral arterial disease is critical evidence of more widespread atherothrombotic disease, with substantial risks of subsequent cardiovascular events and death. Given that the majority has additional comorbidities, these risks are further increased.

## Background

Atherothrombosis is a chronic affliction of the arterial vascular tree. While its coronary and intracerebral manifestations are well recognized, its effects on the peripheral vasculature and the ensuing complications are less so. This condition, peripheral arterial disease, is best known for the ischemic pain it produces, but significant impairment can exist before it is symptomatic. Estimates of the prevalence of peripheral arterial disease vary widely, from 4.3% to 57%, depending on how the disease is identified, the age and risk factor distributions [1-6]. Advancing age, male gender, a history of diabetes and smoking are associated with a higher risk of peripheral arterial disease [7,8]. Approximately one-quarter to more than one-half of patients diagnosed with peripheral arterial disease experience the frequently painful symptoms associated with this diagnosis. Rates of newly diagnosed disease are in the range of 7% to 13% per year [1,7-10].

Awareness and understanding of the significance of a peripheral arterial disease diagnosis is increasing, but precise quantitative estimates of the burden and our understanding of the prognosis and impact of other risk factors remains limited [11]. Documentation of the burden of this disease can provide the means to better understand its course and, in turn, to make more effective decisions in the management of patients. In this paper, we report the results of analyses of the morbidity and mortality experienced by a large cohort of patients diagnosed with peripheral arterial disease.

## Methods

### Study population

The health care databases maintained by Saskatchewan Health were the source of data for this study. Saskatchewan Health is a provincial government department that administers a universal public health insurance program for approximately one million residents. Its administrative databases (e.g., formulary outpatient prescriptions, physician services, hospitalizations, and vital statistics) can be linked electronically using unique patient identifiers [12].

Residents of Saskatchewan, Canada, covered by Saskatchewan Health and eligible for outpatient prescription drug benefits (the prescription drug plan does not capture the claims of Registered Canadians of First Nations), diagnosed with peripheral arterial disease between January 1, 1985 and December31, 1995, during a hospitalization or physician consultation were eligible if at least 21 years of age at the time of diagnosis; no other exclusion criteria were specified. Patients with peripheral arterial disease were identified using the International Classification of Diseases (ICD-9) codes 440, 440.2, or 443.9. The broader three-digit code 443 was allowed if it coincided with documentation of a prescription for pentoxifylline on the assumption that all patients who received this prescription were diagnosed with peripheral arterial disease [13]. Patient history was available from January 1, 1980 and follow-up through March 1998 or until a patient could no longer be followed due to emigration or death. The date of first diagnosis, either hospital admission or physician visit depending on where the index diagnosis was recorded, was taken as the date of entry into the study.

### Reference populations

Analyses were also carried out in reference populations comprised separately of Saskatchewan resident's age 21 years and older, without preexisting peripheral arterial disease, who suffered a myocardial infarction (ICD-9 code 410) or stroke (ICD-9 codes 433, 434, 436, and 362.3) between January 1, 1990 and December31, 1995. The same selection criteria used to identify the subjects with peripheral arterial disease were applied to these populations. The first diagnosis or event on record within the study dates was used to define the populations. For example, a subject with a diagnosis of peripheral arterial disease in October 1988 and a subsequent myocardial infarction recorded in January 1990 was assigned to the peripheral arterial disease cohort.

### Outcomes and risk factors

Hospitalization records subsequent to the index diagnosis were used to identify outcome events, namely angina, transient ischemic attack, myocardial infarction, or stroke. To estimate the event rates, each event was considered independent of the others. The hospital admission date was taken as the time the outcome took place. Deaths were determined from the vital statistics file.

For subjects whose index event was a myocardial infarction, subsequent hospitalizations for myocardial infarction were only considered if they occurred at least seven days after the index event. Similarly, recurrence of stroke had to be at least seven days after an index stroke. For the outcome cluster consisting of angina, transient ischemic attack, myocardial infarction, stroke or cardiovascular death, the first occurrence of any of these events was taken.

Event-free time and survival were measured from the date of the index event (*index date*) to the time of hospitalization for the outcome or the date of death, as appropriate. If no outcome event occurred, then the subject's time was considered right-censored and the study end date (or date of emigration) was taken as the end of the period.

The patients' medical history prior to index event (available back to January 1980) was examined to identify other diseases documented prior to the qualifying diagnosis. Risk factors of interest included: male gender, age greater than 65 years at index diagnosis, prior myocardial infarction, prior stroke, diabetes, hypercholesterolemia, congestive heart failure, angina, atrialfibrillation, hypertension, and transient ischemic attack. The presence of each risk factor was determined based on ICD-9 coding of diagnoses recorded at hospitalizations or doctor visits prior to the index diagnosis. Given the relatively long inclusion period, we also considered the possibility of a calendar trend by including an indicator of the time of diagnosis of peripheral arterial disease, prior to January 1, 1990 or afterwards.

### Analyses

Descriptive analyses were carried out to characterize the study and reference populations in terms of age, gender, setting of diagnosis (hospital versus outpatient), and medical history. The occurrence of each outcome event was independently estimated as a hazard (events/patient years). The Kaplan-Meier approach was used to determine the cumulative proportion (%) of patients suffering *any *one of the outcome events over time. To assess the impact of individual risk factors on the rate of outcome events, Cox proportional hazards analyses were conducted. Next, the number of risk factors present at diagnosis was tallied and the average hazard rates of myocardial infarction, stroke, and death in each group were compared to those of the patients with no risk factors at diagnosis. Comparisons were also made by gender.

Mortality among patients with peripheral arterial disease was evaluated using the Kaplan-Meier method and through estimation of average hazard rates. These were compared to post-acute mortality in each reference population (myocardial infarction and stroke) – deaths recorded in the first 30 days following those acute index events were excluded to accord better with the chronic nature of the diagnoses. The patients' medical history prior to index event (available back to January 1980) was examined to identify other diseases documented prior to the qualifying diagnosis.

## Results

### Population characteristics

The study population consisted of 16,440 patients diagnosed with peripheral arterial disease between 1985 and 1995 with an average follow-up of 5.9 (± 3.6) years. Most patients (90%) were identified on a physician visit and a majority presented with at least one comorbid condition at the time of diagnosis, as displayed in Table 1. Reference populations were comprised of 15,590 patients who suffered a myocardial infarction, 2,700 of whom died during the first 30 days, and 18,704 who suffered a stroke, of whom 1,877 died during the first 30 days. One-third of patients with an index stroke and one-half of patients with an index myocardial infarction were identified in hospital (Table 1).

**Table 1:** **Patient populations**. Patient characteristics and medical history for each population at the time of the index diagnosis. Age is reported as mean ± standard deviation. All other values are reported as number (%).

		**Reference Populations**	
		
**Item**	**PAD** **N = 16,440**	**MI** ** N = 15,590**	**Stroke** ** N = 18,704**
Age (years)	67.3 ± 9.2	66.9 ± 11.1	70.5 ± 9.9
Male	9,030 (55)	9,955 (64)	9,723 (52)
Qualifying diagnosis in hospital	1,659 (10)	8,954 (56)	6,733 (36)
Medical History			
Atrial Fibrillation	862 (5)	905 (6)	1,812 (10)
Angina	5,446 (33)	6,435 (41)	4,849 (26)
Congestive Heart Failure	4,178 (25)	3,902 (25)	5,324 (29)
Diabetes Mellitus	3,172 (19)	3,693 (24)	4,487 (24)
Hypercholesterolemia	1,128 (7)	1,498 (10)	1,183 (6)
Hypertension	9,554 (58)	9,502 (61)	12,603 (67)
Ischemic Stroke	2,111 (13)	1,001 (6)	4,408 (24)
Myocardial Infarction	2,868 (18)	3,672 (24)	2,211 (12)
Transient Ischemic Attack	2,217 (14)	1,372 (9)	5,151 (28)

### Outcome events

Subsequent myocardial infarction, stroke, and angina each occurred in approximately 10% of the peripheral arterial disease population at rates varying between 16.8 and 17.1 per 1,000 patient years (Table 2). Transient ischemic attack occurred in only 3.7% of these patients at a rate of 6.3 per 1,000 patient years.

**Table 2:** **Event risk**. First cardiovascular events of each type and all-cause deaths subsequent to a diagnosis of peripheral arterial disease. Event rates are reported per 1,000 person-years.

	**All Patients**			**Males**			**Females**		
			
**Outcome**	**Events (%)**	**PY**	**Event Rate**	**Events (%)**	**PY**	**Event Rate**	**Events (%)**	**PY**	**Event Rate**
Angina	1,555 (9.5)	90,734	17.1	1,012 (11.2)	48,363	20.9	543 (7.3)	42,371	12.8
TIA	601 (3.7)	94,768	6.3	306 (3.4)	51,526	5.9	295 (4.0)	43,242	6.8
MI	1,563 (9.5)	93,160	16.8	1,012 (11.2)	49,956	20.3	551 (7.4)	43,204	12.8
Stroke	1,589 (9.7)	93,066	17.1	917 (10.2)	50,309	18.2	672 (9.1)	42,757	15.7
Death	7,973 (48.5)	96,771	82.4	4,519 (50.0)	52,503	86.1	3,454 (46.6)	44,269	78.0

**Figure 1:**
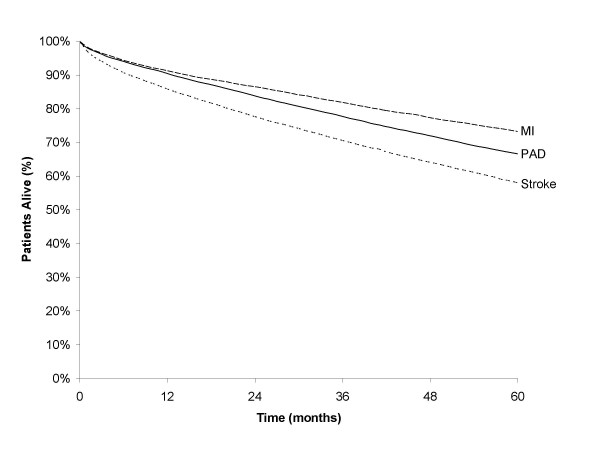
**Patient survival. **Survival among patients with peripheral arterial disease compared to patients suffering a stroke or myocardial infarction.

Only half of all patients remained alive at the end of follow-up. The crude five-year death rate among patients diagnosed with peripheral arterial disease was 33.2% – a rate of 82.4 deaths per 1,000 patient years (Figure 1). When all outcome events and cardiovascular deaths are evaluated together, it is estimated that 16.0% of patients had an event by the end of the first year after diagnosis and this rose to about one-third by three years. In comparison, 13.5% of patients in the myocardial infarction reference population were re-hospitalized for a recurrent myocardial infarction, at a rate of 41.2 per 1,000 person-years, and 12.0% with angina (36.3 per 1,000 person years). Less than 5% suffered a stroke (13.0 per 1,000 person years) or a transient ischemic attack (4.6 per 1,000 person years). Among patients in the stroke reference group, less than 5% suffered a myocardial infarction (12.4 per 1,000 person years), transient ischemic attack (11.1 per 1,000 person years) or angina (7.3 per 1,000 person years), whereas, 16.6% had a recurrent stroke (54.7 per 1,000 person years). Approximately two-thirds (59.6%) of patients with a myocardial infarction and one-half (51.3%) of those with a stroke were alive at the end of follow-up.

### Mortality

The crude five-year death rate among patients diagnosed with peripheral arterial disease was 33.2%. When adjusted for duration of follow-up, the rate is 82.4 deaths per 1,000 patient years. Compared to the reference populations, mortality is lower than among patients who suffer an index stroke (41.8% crude rate; 113.1 per 1,000 patient years adjusted) but higher than that among patients who suffer an index myocardial infarction (26.6%; 63.2 per 1,000 patient years). Survival in each group, excluding deaths during the acute period (first 30 days) for index myocardial infarction and index stroke, is displayed in Figure 1.

### Impact of risk factors

On average, women were older than the men (68.2 ± 8.9 vs 66.5 ± 9.4). Over the course of an average follow-up of six years, men were considerably more likely to manifest further atherothrombotic complications (Table 2): angina (63% more in men), myocardial infarction (59% more), stroke (16% more), and death (10% more). Transient ischemic attack, however, occurred less often in men (13% less).

As might be expected, prior myocardial infarction (hazard ratio = 1.53, 95% CI 1.35–1.73) was a strong risk factor for a subsequent myocardial infarction. A prior stroke was also a predictor of myocardial infarction (hazard ratio = 1.26, 1.08–1.46) and, more strongly, of subsequent stroke (hazardratio = 2.23, 1.96–2.53). In addition, a transient ischemic attack (hazard ratio = 1.35, 1.18–1.54) was a powerful predictor of stroke. Even amongst patients with no history of atherothrombotic disease (myocardial infarction or stroke), however, 7.5% suffered a myocardial infarction and 8.0% a stroke – rates of 13.5 and 14.0 events per 1,000 patient years, respectively.

Cox proportional hazards analyses conducted to determine the effect of various potential risk factors show that the majority of the eleven considered are significant predictors of future stroke, myocardial infarction and death. Apart from prior myocardial infarction or stroke, the risk of myocardial infarction was significantly increased with male gender (hazard ratio = 1.53, 1.38–1.71), age over 65 years (hazard ratio = 1.50, 1.34–1.69), angina (hazard ratio = 1.47, 1.31–1.64), diabetes mellitus (hazard ratio = 1.50, 1.33–1.68), heart failure (hazard ratio = 1.15, 1.02–1.30), and hypertension (hazard ratio = 1.31, 1.18–1.46). Many of these same conditions also significantly increased the risk of stroke: male gender (hazard ratio = 1.26, 1.13–1.39), age over 65 years (hazard ratio = 1.98, 1.73–2.26), atrial fibrillation (hazard ratio = 1.64, 1.36–1.98), diabetes mellitus (hazard ratio = 1.50, 1.34–1.69), heart failure (hazard ratio = 1.20, 1.07–1.35), and hypertension (hazard ratio = 1.51, 1.35–1.69). Increased risk of death was associated with male gender (hazard ratio = 1.27, 1.21–1.32), age over 65 years (hazardratio = 3.65, 3.39–3.92), atrial fibrillation (hazard ratio = 1.25, 1.15–1.35), diabetes mellitus (hazard ratio = 1.40, 1.32–1.47), heart failure (hazard ratio = 2.06, 1.97–2.17), and prior stroke(hazard ratio = 1.53, 1.44–1.62).

When myocardial infarction, stroke, angina, transient ischemic attack, and cardiovascular death are evaluated as a cluster endpoint, 10 of the 11 risk factors were significantly predictive (p ≤ 0.0006) of future events: male gender (hazard ratio = 1.29, 1.23–1.36), age over 65 years (hazard ratio = 1.78, 1.67–1.89), atrial fibrillation (hazard ratio = 1.24, 1.13–1.36), angina (hazard ratio = 1.25, 1.19–1.32), diabetes mellitus (hazard ratio = 1.35, 1.28–1.43), heart failure (hazard ratio = 1.59, 1.51–1.68), hypertension (hazard ratio = 1.11, 1.06–1.17), prior myocardial infarction (hazard ratio = 1.27, 1.19–1.35), prior stroke (hazardratio = 1.51, 1.42–1.62), and prior transient ischemic attack (hazard ratio = 1.12, 1.05–1.20).

**Figure 2:**
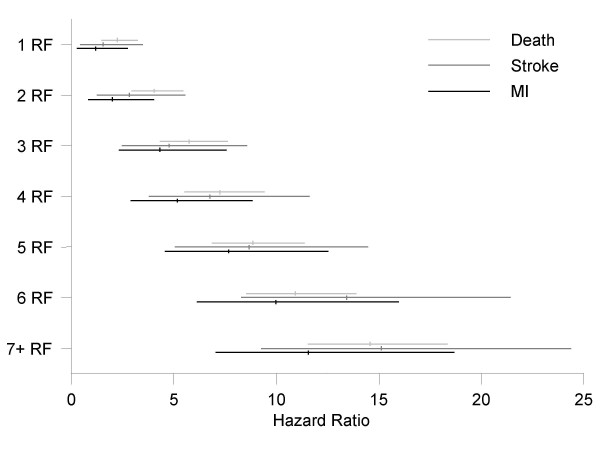
**Relative event risk. **Risk increase in events for various levels of risk factors, compared to no risk factor.

The extent to which the risk increases depends on the number of risk factors present at diagnosis of peripheral arterial disease (Figure 2). Compared to patients with no risk factors at diagnosis (n = 516), presence of one or more risk factors increases the risk of myocardial infarction by 4.08%, of stroke by 5.04% and of death by 5.82%.

To further examine the impact of a prior diagnosis of myocardial infarction or stroke, the 27.7% of patients with at least one of these conditions were evaluated separately. Even in this group, occurrence of a subsequent myocardial infarction was significantly increased with male gender (hazard ratio = 1.57, 1.30–1.90), age over 65 years (hazard ratio = 1.20, 1.00–1.44), angina (hazard ratio = 1.44, 1.21–1.72), diabetes mellitus (hazard ratio = 1.28, 1.06–1.54), and hypertension (hazard ratio = 1.30, 1.09–1.55).

As the study spans more than a decade, the effect of calendar time was examined. Patients diagnosed after January 1, 1990 had an 11.1 % lower risk of subsequent myocardial infarction but there was no effect on stroke or death. Amongst patients with a history of myocardial infarction or stroke, the risk of myocardial infarction was reduced (by 22.3%) for patients with their peripheral arterial disease diagnosed in the 1990s.

## Discussion

Although awareness of peripheral arterial disease and the factors associated with its onset has improved in recent years, to our knowledge, this study provides the first large-scale, long-term comprehensive analyses of the prognosis of patients diagnosed with peripheral arterial diseasetogether with a comparison to those diagnosed first with myocardial infarction or stroke [3-11,14-18]. These analyses expand on current knowledge by providing quantitative estimates of the risks faced by patients diagnosed with peripheral arterial disease and the impact of comorbidities.

A diagnosis of peripheral arterial disease emerged from our analyses as critical evidence of more widespread atherothrombotic disease. The risk of subsequent cardiovascular events is substantial among patients diagnosed with peripheral arterial disease even when prior atherothrombotic disease (myocardial infarction and prior ischemic stroke) is considered. The majority of these patients have additional cardiovascular risk factors at the time of diagnosis, thus contributing to these increased risks.

In comparisons with patients suffering an acute stroke or myocardial infarction, comparable proportions of patients with peripheral arterial disease suffered subsequent events and death. Generally, risks among patients with peripheral arterial disease were observed to be lower than those suffering a stroke and higher than those experiencing a myocardial infarction. Exceptions included higher rates of subsequent myocardial infarction and angina among patients in the myocardial infarction group and lower rates of myocardial infarction and transient ischemic attack among patients in the stroke group. These results suggest that patients diagnosed with peripheral arterial disease are at comparable risks for experiencing subsequent events.

These findings are consistent with those of patients in a six-year follow-up study who were identified as having disease on non-invasive screening measurement of the ankle-brachial index. Approximately half suffered a major acute event (stroke or myocardial infarction) or underwent major surgery (amputation or vascular surgery) within six years of referral to a specialty clinic [17]. Another one quarter of patients died during this period. Over ten years, this estimate is notably higher with 62% of men and 33% of women expiring within this time-more than two times the rate observed in men without a diagnosis of peripheral arterial disease and approximately twice the rate among women [18]. Concomitant diagnoses, such as diabetes mellitus and hypertension were associated with significant risk of morbidity and mortality. As well, atrial fibrillation was linked to an increase in acute emergency admissions and in-hospital mortality in a recent study of patients with symptomatic peripheral arterial disease [19]. Evaluation of the medical treatment received by patients in this cohort was beyond the scope of this study and was not considered in our analyses.

Analyses conducted using administrative data such as those we used from Saskatchewan Health have some limitations. Definition of the populations and identification of events is dependent on accuracy of the ICD-9 codes submitted to Saskatchewan Health. Validation work has indicated, however, a low error rate for cardiovascular diagnoses [12]. Another problem with administrative data is that they do not contain clinical information such as results of laboratory tests, smoking, family history, blood pressure, and so on. Thus, these potentially relevant factors could not be incorporated in the analyses.

## Conclusion

Despite prior data in favor of treating peripheral arterial disease as evidence of disseminated atherothrombosis, the management of patients with this diagnosis remains less aggressive than for patients suffering cardiovascular events [3,20]. The results of this study reinforce the call for attention to peripheral arterial disease as a serious diagnosis with important implications for patient management. In particular, identification of other risk factors and risk reduction through pharmacologic or other management strategies ought to be aggressively pursued in this population, as is already common practice among patients who suffer a myocardial infarction or stroke.

## Abbreviations

CI: Confidence Interval

ICD-9: International Classification of Diseases, version 9

MI: Myocardial Infarction

PAD: Peripheral Arterial Disease

PY: Person-Years

RF: Risk Factors

TIA: Transient Ischemic Attack

## Competing interests

This research was supported in part by an unrestricted grant from Sanofi-Aventis & Bristol-Myers Squibb. Neither funding agency had any role in the design, execution of the analyses, their interpretation, or the writing of the paper. The authors had sole responsibility for all methodological decisions.

## Authors' contributions

JC designed the study, participated in the data analyses and writing of the paper. KMW participated in the study design, data analyses and in writing the paper. KJI. led the analyses and participated in writing the paper. IP conducted the analyses and helped write the paper.

## Pre-publication history

The pre-publication history for this paper can be accessed here:


